# Congenital Defects in a Patient Carrying a Novel Homozygous *AEBP1* Variant: Further Expansion of the Phenotypic Spectrum of Ehlers–Danlos Syndrome Classical-like Type 2?

**DOI:** 10.3390/genes13122358

**Published:** 2022-12-14

**Authors:** Niccolò Di Giosaffatte, Alessandro Ferraris, Federica Gaudioso, Valentina Lodato, Emanuele Savino, Claudia Celletti, Filippo Camerota, Simone Bargiacchi, Luigi Laino, Silvia Majore, Irene Bottillo, Paola Grammatico

**Affiliations:** 1 Laboratory of Medical Genetics, Department of Experimental Medicine, San Camillo-Forlanini Hospital, Sapienza University, 00185 Rome, Italy; 2 Physical Medicine and Rehabilitation Division, Umberto I University Hospital of Rome, 00161 Rome, Italy

**Keywords:** Ehlers–Danlos Syndrome, *AEBP1*, ACLP, clEDS2, multiple congenital anomalies, TGF-β pathway, cleft palate, amniotic band sequence, Poland anomaly

## Abstract

In 2018, a new clinical subtype, caused by biallelic variants in the *AEBP1* gene, encoding the ACLP protein, was added to the current nosological classification of the Ehlers–Danlos Syndromes (EDS). This new phenotype, provisionally termed EDS classical-like type 2 (clEDS2), has not yet been fully characterized, as only nine cases have been reported to date. Here we describe a patient, homozygous for a novel *AEBP1* pathogenic variant (NM_001129.5 c.2123_2124delTG (p.Val708AlafsTer5)), whose phenotype is reminiscent of classical EDS but also includes previously unreported multiple congenital malformations. Furthermore, we briefly summarize the current principal clinical manifestations of clEDS2 and the molecular evidence surrounding the role of *AEBP1* in the context of extracellular matrix homeostasis and connective tissue development. Although a different coexisting etiology for the multiple congenital malformations of our patient cannot be formally excluded, the emerging role of ACLP in TGF-β and WNT pathways may explain their occurrence and the phenotypical variability of clEDS2.

## 1. Introduction

The term Ehlers–Danlos syndrome (EDS) represents a group of hereditary connective tissue disorders (HCTD) caused by perturbations of the function and structure of the extracellular matrix (ECM). Common clinical manifestations of these disorders are generalized joint hypermobility, skin hyperextensibility and soft tissue fragility, especially affecting the skin, the joints, and the blood vessels.

The most recent classification (dated 2017) recognized 13 different clinical subtypes of EDS [1]. Each subtype is designated with a descriptive term, usually representative of the most striking manifestation or affected district. Major and minor diagnostic criteria were proposed for each subtype based on specific or more frequent features [1]. This wide group of conditions have been associated with pathogenic variants in 20 genes implicated in collagen structure, processing, and folding, in glycosaminoglycan biosynthesis or in the maintenance of ECM mechanical properties [2]. The commonest form of EDS is the hypermobile type (hEDS), which so far has not been associated with any major causative genes. Two other well-known less frequent types are the classical EDS (cEDS) and the vascular EDS (vEDS), mostly due to heterozygous pathogenic variants in *COL5A1/COL5A2* and *COL3A1* genes, respectively. These disorders are recognized as having autosomal dominant mendelian inheritance. Conversely, 7 out of 10 other ultrarare subtypes follow an autosomal recessive inheritance. Among them, the classical-like EDS type 1 (clEDS1) is caused by biallelic mutations in the *TNXB* gene. 

In 2016, Alazami and colleagues reported two siblings with an EDS-like phenotype, who were found to be homozygous carriers of a pathogenic variant in the adipocyte enhancer binding protein 1 (*AEBP1*) gene [3]. Considering the clinical appearance, partially resembling cEDS, and the autosomal recessive inheritance, this condition was provisory named classical-like type 2 EDS (clEDS2, MIM #618000). Since this first report, nine additional patients from seven unrelated families have been described to date [4,5,6,7]. 

The *AEBP1* gene includes 21 exons and is actually transcribed by alternative splicing in two mRNA variants that are translated in one shorter AEBP1 protein with nuclear localization, and one longer isoform denominated ACLP (acronym for aortic carboxypeptidase-like protein) [8]. ACLP includes an N-peptide signal for molecular trafficking in the endoplasmic reticulum for post-translational modifications and it is secreted in the extracellular space [9]. Both histological and functional studies have been performed to investigate the role of *AEBP1* in an ECM context. Histological and ultrastructural analyses conducted on clEDS2 patients demonstrated decreased dermal collagen [4] and variation in diameter sizes of collagen fibrils, which in addition presented with a frayed/ragged moth-eaten appearance and a collagen flowers organization in horizontal and longitudinal sections, respectively [4,7]. At a functional level, ACLP has been demonstrated to bind collagen type I, type III, and type V [4] and to enhance the stiffness, toughness, and tensile strength of COL1 fibers in vitro [9].

Due to the novelty of clEDS2, no clinical criteria have been proposed yet. The reported cases resembled cEDS, showing generalized joint hypermobility, skin hyperextensibility, and atrophic scarring, but a broader and more variable phenotype for clEDS2 has been noticed [7]. In this report, we describe a novel case of clEDS2 presenting, in addition to the typical EDS features, multiple congenital defects, further expanding this variability. We then proceed to compare this patient with previously reported cases and discuss how his peculiar clinical findings might have occurred in the context of the dysregulation of critical pathways caused by a ACLP deficiency.

## 2. Materials and Methods

### 2.1. Patient

The proband was referred to our clinic at San Camillo-Forlanini Hospital/“Sapienza” University of Rome, for an evaluation for a suspected HCTD. A full clinical examination and a revision of previous medical records and investigations were performed. Due to the complex phenotype, combining EDS features with multiple congenital defects, such as cleft palate, foot malformation, and agenesis/hypoplasia of the right pectoral muscle, whole exome sequencing (WES) and cytogenomic characterization was requested. 

### 2.2. Genetic Analyses

Whole exome sequencing (WES) was performed on genomic DNA extracted from peripheral blood through a Nextera DNA Exome kit (Illumina, San Diego, CA, USA) using a NextSeq2000 sequencer (Illumina). Sequencing reads were aligned to the human reference genome (UCSC hg19) by the BWA software package (v0.7.7-isis-1.0.2) (Illumina). Variant calling was performed by the GATK Variant Caller (v1.6-23-gf0210b3). The DNA variants were annotated by eVai (v.2.6) (EnGenome). After filtering by MAF < 0.01 (GnomAD v2.1), variants mapping in genes associated to the following human phenotype ontology (HPO) phenotypes were prioritized: HP:0001382 (joint hypermobility), HP:0002761 (generalized joint laxity), HP:0001388 (joint laxity), HP:0003549 (abnormality of connective tissue), HP:0000175 (cleft palate), HP:0008953 (pectoralis major hypoplasia), HP: 0010185 (aplasia/hypoplasia of the distal phalanges of the toes), and HP:0000974 (hyperextensible skin). Filtered variants were classified according to ACMG/AMP criteria [10]. Sanger sequencing was employed to validate the pathogenic variant both in the proband and in the DNA from peripheral blood of his parents, by the use of the following PCR primers pair:

AEBP1 Ex17_FW: 5′-CTGAACCCTGATGGCTAC-3′

AEBP1 Ex17_Rv: 5′-CACTATCCCCAACCTAGC-3′

Genomic DNA was extracted from peripheral blood by the standard method. A chromosomal microarray was performed using Illumina Infinium CytoSNP-850K BeadChip v. 1.2 [hg19], according to manufacturer instructions. The BeadChip slide was scanned using a NextSeq550Dx scanner (Illumina). The DNA copy number and loss of heterozygosity (LOH) analyses were performed using BlueFuse Multi Software Edition v.4.5.

## 3. Results

### 3.1. Clinical Findings

The patient was a 26 year old male who was referred to our attention because of generalized joint hypermobility and musculoskeletal pain of the back lasting for 5–6 years. 

#### 3.1.1. Familial and Personal History

He was born from unaffected non-consanguineous parents. Family history was unremarkable for connective tissue disorders, other genetic conditions, and congenital defects. After an uneventful pregnancy, he was born preterm (36 g.w.) due to the spontaneous onset of labor. A C-section was performed at delivery because of podalic presentation. At birth, he required cardiac resuscitation with oxygen support; the APGAR score was 3^1 min^/7^5 min^. The birth weight was 2400 g, and the length was 47 cm. He was admitted to the neonatal pathology department due to perinatal distress, a low birth weight, and multiple congenital defects: a cleft palate and a unilateral distal reduction malformation of the left foot were noticed along with mild facial dysmorphisms (epicanthus, low set ears, and micro/retrognathia). Other reported features during first infancy were mild muscular hypotonia, diastasis recti, and bilateral cryptorchidism with a left inguinal hernia. The patient underwent a surgical correction of the cleft palate and cryptorchidism at age 12 months and 3 years, respectively. Although the developmental milestones were reported to be normally achieved, he attended regular education with support during primary school. The puberal development was regular. Recurrent joint subluxations, skin fragility with easy bruising/multiple hematomas, and a tendency to develop callous lesions of the hands and feet were referred to be present since childhood.

#### 3.1.2. Physical Examination

**Figure 1:**
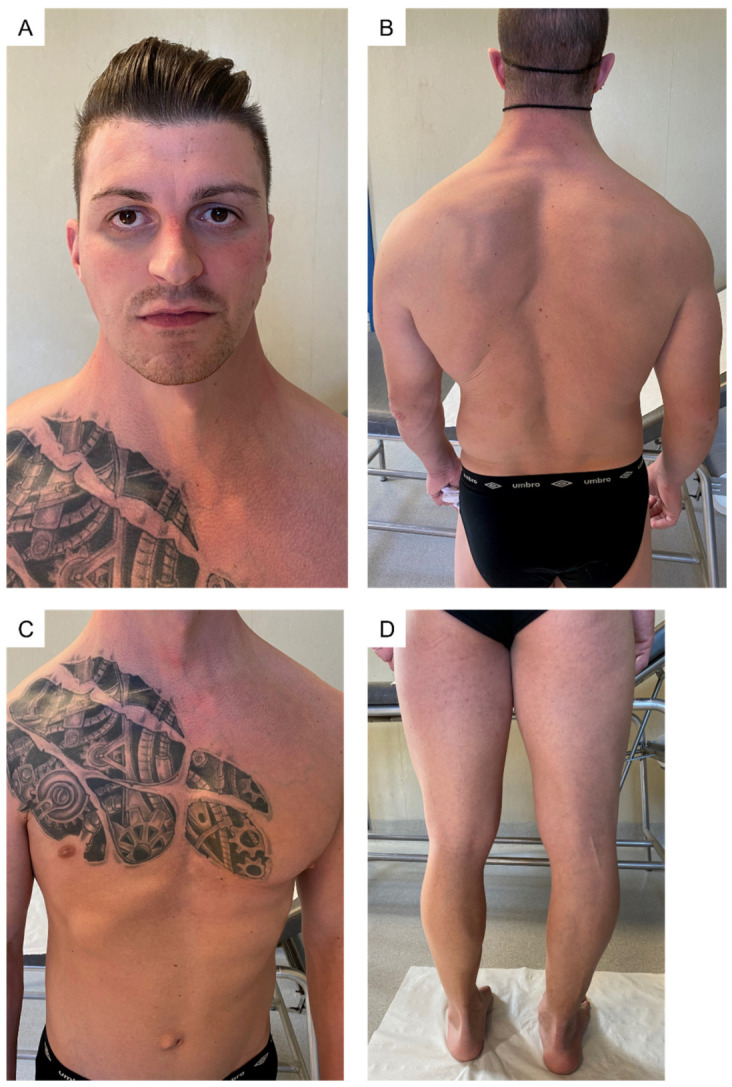
General examination of the patient. (**A**) mild facial dysmorphism (down-slanting palpebral fissures, epicanthus, deep set eyes, malar hypoplasia; (**B**) webbed neck, scoliosis; (**C**) thoracic asymmetry due to unilateral hypoplasia of right pectoralis major muscle; (**D**) lower limbs dysmetria.

On first physical examination at our clinic, biometric measurements were height = 174.5 cm (−0.3 SD); weight = 80 kg; (+0.68 SD) arm span = 180 cm; and AS/h ratio = 1.03. Mild facial dysmorphisms (down slanting palpebral fissures, deep set eyes, malar hypoplasia, and webbed neck) were observed along with an armonic habitus with thoracic asymmetry due to unilateral hypoplasia of right pectoralis major muscle, scoliosis, hips dysmetria and shortening of the left limb (Figure 1).

**Figure 2:**
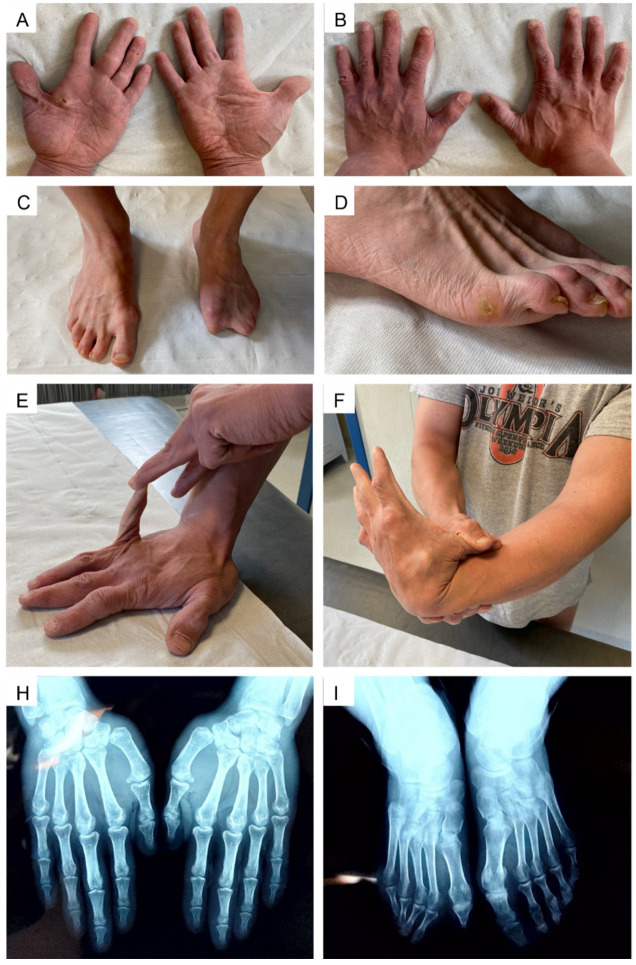
Extremities and joints assessment: (**A**,**B**), progeroid aspect of the hands with apparently short “stubby” fingers; (**C**,**D**) right foot with hammer toes; left foot with aplasia/hypoplasia of phalanges; (**E**,**F**) joint hypermobility of the V and I finger. Beighton score was 7/9, including distal hypermobility. (**H**): radiogram of the hands; (**I**): radiogram of the feet.

The hands presented with a progeroid aspect and short “stubby” fingers with palmar and digital pits. Palmar callosities putatively resulting from mechanical injuries due to reported weight-lifting activity were also evident. The examination of the feet revealed hammer toes on the right side and an apparent absence of the I, IV, and V finger and hypoplasia of the II and III finger of the left foot. Generalized joint hypermobility was noticed since the Beighton score was 7/9 (range of motion beyond the normal limits at knees, back, and the I and V finger on both hands); hyperextensibility of the interphalangeal and metacarpophalangeal joints was also observed. Wrist and thumb signs were negative (Figure 2).

**Figure 3:**
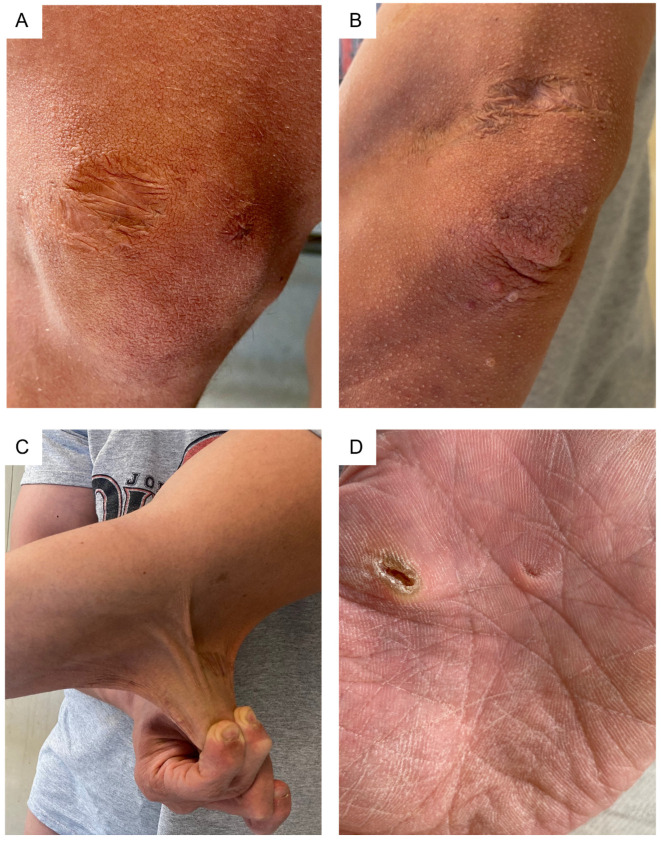
Skin examination: (**A**,**B**) atrophic and hemosiderotic scars of the knee and elbow, the latter also showing small molluscoid pseudotumors and skin wrinkling/acquired cutis laxa.; (**C**) marked skin hyperextensibility; (**D**) hyperlinear palm with pits, callosities, and apparently aberrant keratinization.

Skin was dry, markedly hyperextensible (>3 cm dorsum of the hand, >5 cm elbow and neck regions), and translucent, with visible vascular architecture, especially around the neck and chest. Some scars were atrophic, with one on the left elbow presenting with subcutaneous spheroids, while others being hemosiderotic or having a cigarette-paper-like appearance. Piezogenic papules were present at the heels (Figure 3). An oral examination identified hypertrophic double frenulum of the upper lip and hypoplasia of the lingual frenulum. 

#### 3.1.3. Instrumental Investigation

Instrumental assessments previously undertaken were evaluated. Radiograms at age 11 showed the presence of scoliosis with right hip and left shoulder dysmetria. Radiograms on the left foot at six months documented cutaneous webbing, an absence of the intermediate and distal phalanges of the II to V toes, an absence of the distal and dysplasia of the proximal phalanges of the I toe. Another radiograph at age 26 confirmed the anomalies on the left foot, additionally describing the contralateral valgus metatarsophalangeal joint of the I toe with arthrosis. From panoramic dental X-rays, multiple dental caries and diffuse signs of periodontal disease were identified. Echocardiograms at age 21 showed systolic arching of the anterior mitral leaflet with mild regurgitation at tricuspid, pulmonary, and mitral valves with no hemodynamic relevance. The bone densitometry at 26 years was within normal limits.

### 3.2. Genetic Findings

**Figure 4:**
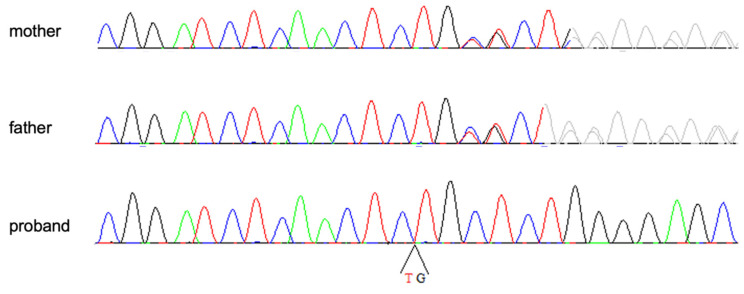
Electropherograms of the homozygous patient and his heterozygous parents.

A cytogenomic analysis did not detect any significant chromosomal imbalance, while the WES study revealed the presence of the homozygous variant NM_001129.5:c.2123_2124delTG (NP_001120.3:p.Val708AlafsTer5) in exon 17 of the *AEBP1* gene. The variant is rare, not being observed in the large population database GnomAD (v2.1.1, https://gnomad.broadinstitute.org, accessed on 30 November 2022), and it is predicted to alter protein translation by creating a premature stop codon that is predicted to trigger nonsense mRNA mediated decay. For these reasons, and the correspondence of clinical features with those reported in clEDS2, the variant was then classified as pathogenic. Sanger sequencing confirmed the variant in homozygosity in the proband and demonstrated its presence in heterozygosity in both unaffected parents (Figure 4).

The DNA variants resulting from NGS data analysis and filtering, as well as their ACMG/AMP classification, are reported in Supplementary Table S1.

## 4. Discussion

We reported a novel homozygous *AEBP1* pathogenic variant in a patient affected by Ehlers–Danlos Syndrome and multiple congenital defects previously unnotified in the classical-like 2 EDS subtype. As shown by Table 1, our patient presented all major clinical features reported in other clEDS2 cases, with the exception of osteoporosis, possibly due to the young age, ocular anomalies, and delayed wound healing. The clEDS2 phenotype has previously been compared by other authors to other types of EDS, such as classical EDS, vascular EDS and arthrochalasia EDS [4], cardiac valvular, hypermobile, and kyphoscoliotic EDS [5], and spondylodysplastic EDS [7]. This overlap with more than one of the other EDS subtypes reflects the highly variable presentation of clEDS2. Such phenotypical heterogeneity, acknowledged by all of the authors, complicates the formalization of clinical suggestive criteria for this EDS subtype. However, it seems that, at the present time, the clinical criteria suggestive for clEDS2 could be the presence of major criteria for classical EDS (i.e., skin hyperextensibility and atrophic scarring together with generalized joint hypermobility), accompanied by at least two “diagnostic handles” among foot deformities (i.e., hallux valgus, hammer toes, pes planus, hindfoot deformities), hair anomalies (alopecia, progressive thinning of the hair), progeroid aspect of hands or feet, and early onset osteopenia/osteoporosis. These clinical findings can also be supported by an apparent autosomal recessive mode of inheritance.

Despite his peculiar phenotype, the patient waited 26 years before receiving a genetic diagnosis and consequently entering a dedicated clinical follow up, also being counselled about the risk for complications related to EDS. Since no etiological therapies for this type of disorder are available, treatments and preventive measures for complications can only be symptomatic and should be pursued in a multidisciplinary setting. Specifically, chronic pain and articular instability should be followed up and treated by specialists in physical medicine, orthopedics, and pain management, while skin features should be managed by a dermatologist. Moreover, the surveillance and prevention of early onset osteoporosis requires periodic evaluations through bone densitometry, biochemical tests of bone metabolism markers, and therapies with supplements and dedicated drugs as needed. The achievement of the diagnosis, in addition to having guided the organization of the patient’s clinical management, also allowed him to receive genetic counseling about the recurrence risk of the disorder in his future offspring. Given the autosomal recessive inheritance, it will be possible to offer a carrier test to the partner to estimate the reproductive risk; if this test will result in being positive, prenatal genetic testing through invasive or non-invasive options [11] will be discussed with the couple.

To date, multiple congenital defects have not been reported in patients with clEDS type 2. Due to the paucity of cases, it is worth questioning if, in the present subject, the co-occurrence of cleft palate, hypoplasia/aplasia of multiple distal bones of the left foot, and the disruption of the right pectoral muscle could be coincidental or might be related to the etiopathogenetic process leading to clEDS2. In favor of the latter hypothesis, no acquired causes, such as maternal exposure to teratogenic or disruptive agents, were reported during the pregnancy. Moreover, a cytogenomic analysis did not reveal any relevant genomic microdeletion or microduplication, and a WES analysis did not find any clinically relevant variant in genes known to be associated with malformative syndromes. Thus, even though an independent concomitant multifactorial etiology cannot be completely excluded, it is tempting to assume that the congenital defects of the described patient could also be related to the presence of the identified homozygous *AEBP1* pathogenic variant.

When addressing a congenital defect, it is of utmost importance to investigate which pathogenic mechanism between malformation, disruption, and dysplasia is responsible for its occurrence. The right pectoralis muscle hypoplasia observed in the present case resembled what is found in the congenital absence of a pectoralis muscle (also known as the Poland anomaly, MIM %173800), thus suggesting a disruptive sequence. Contextually, the left distal limb defects are reminiscent of an amniotic band sequence (ABS). Even occurring rarely, ABS has been associated with EDS, particularly with the vascular subtype having been reported in 5 out of 1232 cases (0.4%) [12] and, more recently, in 7 out 411 cases (1.7%) [13] indicating an increased risk at of least six times greater than in the general population [12]. Moreover, among the other rare HCTDs, ABS has also been described in two carriers of the COL1A2 mutation affected by osteogenesis imperfecta [14,15]. The exact pathogenesis of ABS is still a matter of debate and two main hypotheses have been proposed. According to the mostly accepted extrinsic hypothesis, following the rupture of the amnion, the floating amniotic bands wrap the distal part of the limbs, exerting an external mechanical compression, thus perturbing the angiogenetic and developmental processes. Chorioamniotic membranes have a fetal origin and collagen type III and type I are two of its major constituents, together with type V. Thus, some authors suggest that the perturbation of collagens during the development of amniotic membranes in fetuses heterozygous for the COL3A1 mutation might lead to an increased propensity for amnion damage, thus increasing the risk of ABS [13,16]. Conversely, since some ABS cases in literature do not entirely fit the aforementioned extrinsic hypothesis, other authors suggested an intrinsic hypothesis owing to primary vascular anomalies with a subsequent occurrence of limb defects [17]. In other words, the occurrence of limb defects could be driven by a primary failure of the molecular program responsible for vascular development and homeostasis. Interestingly, considering this latter theory, some evidence from literature shows that ACLP activity is associated with relevant developmental molecular pathways, specifically those of WNT and TGF-β [18,19].

In human fibroblast culture assays, ACLP has been shown to positively modulate the TGF-β pathway through the binding of TGF-β receptor II (TbR-II). This interaction probably facilitates the formation of the tetrameric TbR-II/TbR-I receptor complex which, after the binding of TGF-β in its activated form, induces Smad2/3 phosphorylation [19]. The TGF-β pathway has a major role in the development and homeostasis of connective tissues, contributing to vessel wall structure, limb and digit development, the fusion of the soft palate, endochondral ossification, longitudinal bone growth, and bone remodeling [20]. The presence of a cleft palate suggests that, in the present *AEBP1*/ACLP mutated patient, a perturbation of the TGF-β pathway could be the cause of this congenital defect. Furthermore, other clinical features reported in our and other clEDS2 cases are frequently found in Loeys–Dietz syndrome, a hereditary condition caused by heterozygous mutation in one gene among *TFGBR1*, *TGFBR2*, *TGFB2*, *TGFB3*, *SMAD2*, and *SMAD3*. Examples of such overlapping features are facial dysmorphisms, such as down-slanting palpebral fissures, midface hypoplasia/retrognathia, (present case, B-II [4]; IV-6 andIV-6-2 [3], Pt.1 [9]), a high arched palate (Pt.1 [4], IV-6 [3]), aortic dilation (B-II [4]), arachnodactyly (D-II:1 [5]), positive wrist and thumb signs (D-II:1 [5]), and osteoarthritis/early onset arthrosis (A-II; B-II:1 [4], D-II:1 [5]). Since ACLP seems to act in the TGF-β pathway only as a positive modulator, the final effect of its deficiency could probably also be influenced by other regulators of this pathway, thus producing variable outcomes among different affected subjects. This could be one of the possible factors explaining the phenotypical variability in clEDS2. This consideration might also be applied to congenital defects, such as the Poland anomaly and ABS, in which the perturbation of the TGF-β pathway may not be sufficient to cause the defect but may represent a risk factor for their occurrence. More studies are needed to support this hypothesis.

## 5. Conclusions

We described a patient affected by clEDS2 caused by a novel homozygous pathogenic variant in the *AEBP1* gene. The most frequent clinical manifestations of reported cases together with the evidence of the role for ACLP in the context of ECM justify the classification of the phenotype of *AEBP1* pathogenic variant carriers among the EDS subtypes. In addition, ACLP seems to have an additional role as a modulator of the TGF-β pathway. The congenital defects presented here, together with other minor clinical features, could represent clues to emphasize a possible link between clEDS2 and the perturbation of this pathway. Since the number of reported patients is still too low to rule out an independent cause for these additional clinical features, more clinical reports are needed together with studies aimed to investigate the presence of aberrant TGF-β signaling in clEDS2.

## Figures and Tables

**Table 1:** clEDS2 reported clinical features in this report and their overall frequencies in published cases.

Clinical Features	Reported in	Features of this Case
**Mucocutaneous Features**		
Cutaneous hyperextensibility	10/10	+
Easy bruising	10/10	+
Redundant skin	8/10	+
Delayed wound healing	8/10	−
Atrophic scarring	7/10	+
Translucent skin	6/10	+
Progeroid aspect hands/feet	6/10	+
Helicitable piezogenic pedal papules	4/10	+
Paradontal/tooth anomalies ^a^	4/10	+
Hair anomalies ^b^	4/10	−
Soft doughy texture	2/10	−
**Musculoskeletal features**		
Hypermobility ^c^	9/10	+
Foot deformities ^d^	10/10	+
Dislocation/subluxations ^e^	9/10	+
Scoliosis/kyphosis	7/10	+
Other anomalies of the spine ^f^	4/10	−
Osteopenia/osteoporosis	5/10	−
Early onset arthrosis ^g^	4/10	−
Pectus excavatum	3/10	−
Inguinal/umbilical hernias	4/10	+
Muscular involvement ^h^	3/10	+
Perinatal hypotonia	3/10	+
**Cardiovascular Features**		
Cardiac valves anomalies ^i^	5/10	+
Hematomas	3/10	*+*
Varicose veins	3/10	*+*
Postural orthostatic tachycardia syndrome	2/10	−
**Other Features**		
Preterm/premature birth	5/10	+
Bilateral cryptorchidism	3/6	+
Ocular anomalies ^j^	5/10	−
Mild facial dysmorphism ^k^	5/10	+
Delays motor development	5/10	+

^a^ Periodontal disease (2), bad tooth qualities (enamel defects), and multiple caries (3); ^b^ alopecia (3), thinning of the hair (2); ^c^ generalized joint hypermobility (9), localized hypermobility (1) reported only in patient D-II in whom BS was assessed at age 38 (see reference [5]); ^d^ hallux valgus (6), hammer toes (5), pes planus (8), hindfoot deformities (2), aplasia/hypoplasia of the distal phalanges of the feet digits (1); ^e^ shoulder (7), knees (4), elbow (3), wrist (2), hip (3), ankle (3), finger (2); ^f^ vertebral anomalies (3), disc bulging (2); ^g^ spine (2), knee (1), temporomandibular (1); ^h^ muscle weakness (2), right pectoralis hypoplasia (1); ^i^ 3 mitral valve prolapse, 2 mild polivalvular insufficiency; ^j^ myopia (4), astigmatism (3), keratoconus (1); ^k^ micrognathia (4), webbed neck (2), low set ears (2)**.**

## Data Availability

The data presented in this study are available on request from the corresponding author.

## Supplementary Materials

The following supporting information can be downloaded at: https://www.mdpi.com/article/10.3390/genes13122358/s1, Supplementary Table S1: DNA variants resulting from the NGS data analysis. The table reports, for each variant, the HGVS nomenclature, the ACMG/AMP classification, annotations and quality scores of the variant’s call.
